# The Influence of Hot Isostatic Pressing on the Mechanical Properties of Ti-6Al-4V Samples Printed Using the LENS Method

**DOI:** 10.3390/ma18030612

**Published:** 2025-01-29

**Authors:** Bożena Gzik-Zroska, Kamil Joszko, Agata Piątek, Wojciech Wolański, Edyta Kawlewska, Arkadiusz Szarek, Wojciech Kajzer, Grzegorz Stradomski

**Affiliations:** 1 Department of Biomaterials and Medical Devices Engineering, Faculty of Biomedical Engineering, Silesian University of Technology, 41-800 Zabrze, Poland; wojciech.kajzer@polsl.pl; 2 Department of Biomechatronics, Faculty of Biomedical Engineering, Silesian University of Technology, 41-800 Zabrze, Poland; kamil.joszko@polsl.pl (K.J.); wwolanski@polsl.pl (W.W.); edyta.kawlewska@polsl.pl (E.K.); 3 SKN “Biokreatywni”, Faculty of Biomedical Engineering, Silesian University of Technology, 41-800 Zabrze, Poland; agatpia347@student.polsl.pl; 4 Department of Technology and Automation, Faculty of Mechanical Engineering and Computer Science, Czestochowa University of Technology, 42-200 Częstochowa, Poland; arkadiusz.szarek@pcz.pl; 5 Faculty of Production Engineering and Materials Technology, Czestochowa University of Technology, 42-201 Częstochowa, Poland; grzegorz.stradomski@pcz.pl

**Keywords:** titanium alloy, static tensile test, digital image correlation, 3D printing parameters

## Abstract

The aim of this work was to assess the influence of the parameters of the hot isostatic pressing (HIP) process and the direction of printing of Ti-6Al-4V samples made using the laser-engineered net shaping (LENS) method on strength properties. The tests were carried out using a static testing machine and a digital image correlation system. Samples before and after the HIP process were tested. The HIP process was carried out at a temperature of 1150 °C, a heating time of 240 min and various pressure values of 500, 1000 and 1500 bar. Based on the comparative analysis of the test results, it has been shown that the ability to adjust the parameters of the HIP process has a significant impact on the final mechanical properties of the samples.

## 1. Introduction

**Figure 1:**
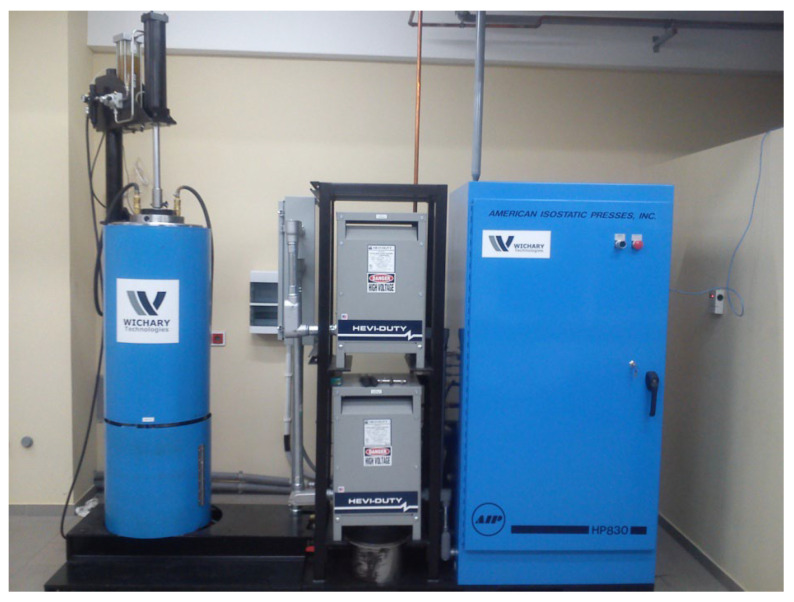
High-temperature isostatic press HIP AIP8-30H-PED.

Additive technologies, which have been developing very dynamically in recent years, have their origins in rapid prototyping techniques. These technologies date back to the 1980s. Various types of rapid prototyping have been introduced since the Cold War for the development of advanced military industry and space technology. Depending on their purpose, they would be likely to be implemented; choosing of materials has become a sensitive issue with a lot of challenges. Many improvements have been made since then, but the idea itself is still based on the same assumptions. The early application of these solutions was very closely linked to foundry engineering, and the main task was to obtain a 3D model to assess manufacturability. Since then, the development of laser technology, electronics, computer science and also material engineering has allowed the creation of fully fledged products using 3D printing technology. Figure 1 shows such an example. However, 3D printing technology itself has its drawbacks, in addition to its huge advantages. Therefore, for some applications, especially those where the printed element is to be used, additional finishing processing is necessary. One such technique, the task of which is to change the functional properties of the finished product, is heat pressure processing [1,2,3].

The isostatic pressing process is an advanced material processing method used in industry to produce metal parts with high durability. This method involves compressing the material in a chamber using the appropriate temperature and pressure [4,5].

Based on the appropriately selected temperature and pressure, the isostatic pressing process is divided into a high-temperature process, using high temperatures in the range of 1200–2200 °C and high isostatic pressure for processing [6,7], and a low-temperature process, using high hydrostatic pressure and an ambient temperature.

Hot isostatic pressing (HIP) is a technology that, in general, improves the mechanical properties of the processed material; however, this technique can also degrease mechanical properties [7,8]. It should be remembered that the interaction of pressure and temperature is very complex. The HIP process closes the porosity, but may also lead to grain growth, which in most cases will negatively affect some mechanical properties. Many works by previously un-published authors indicated the possibility of a decrease in the tensile strength of the material after the HIP process, but at the same time, an increase in fatigue or impact resistance was observed. This is why it is so important to correctly define the scope that is required and what functional properties are crucial.

Its main advantage is the ability to remove internal porosity in castings, elements printed using additive methods or obtained by metal injection. The hot isostatic pressing process takes place in a special, tightly closed vessel, which is placed in an appropriate furnace. The vessel with the sample inside is subjected to high temperature and high isostatic pressure. The pressure is constant throughout the vessel and affects the material from every direction. In a material that is subjected to elevated temperature and high pressure, phenomena such as plastic deformation, creep and diffusion occur. Both factors are kept constant, which allows the material to form evenly. High pressure affects changes in the material’s phases and interphase reactions, while increased temperature allows it to become flexible. Changes in the structure of the processed material are irreversible. The HIP process also aims to reduce internal stresses to avoid micro-cracks that could lead to serious damage to the material [4,5,6]. The isostatic pressing process is becoming increasingly popular among engineers. Research is being carried out to determine changes in the properties of samples subjected to heat treatment, such as isostatic pressing. The research methodology used differs in terms of the materials tested, the method of preparing samples for testing and the parameters of the isostatic pressing process [7].

The process of isostatic pressing and shaping the geometry of surfaces by 3D printing can also be used in biomedical engineering. Currently, medical implants are mainly manufactured from titanium alloys, which have a favorable set of mechanical properties (apart from insufficient resistance to abrasive wear), a high degree of biocompatibility and positive bioactivity in relation to the surrounding cellular structures. Biomaterials of this type are characterized by high specific strength and relatively low (favorable) stiffness, and the favorable surface properties in terms of biotolerance are obtained thanks to a naturally formed, compact oxide layer. The phenomena of biological integration at the implant-plant–tissue interface, apart from medical factors, depends on so-called primary stabilization. The best results in this respect are obtained by appropriately shaping the implant in terms of geometry and surface topography in such a way that it is possible to mechanically anchor the implant in the surrounding tissues. The quality and condition of the implant’s surface layer has a direct impact on the ability to structurally and functionally connect the “used” implant with living tissue [9,10].

The presented arguments encourage a deeper analysis of the problem of modification in terms of the geometry and topography of the surface layer. This possibility is provided by, among others, advanced additive techniques, which not only create new opportunities for the surface modification of existing medical implants, but also enable the concurrent shaping of geometry (adapted to the patient’s individual anatomical features) and structures with increased biofunctionality for medical implants [11,12,13,14]. One of the more promising, innovative manufacturing technologies in this respect is the laser-engineered net shaping (LENS) method. This enables, unlike techniques from the selective laser melting (SLM) and electron beam melting (EBM) groups, the implementation of hybrid manufacturing processes, e.g., shaping the final geometry on a core with a simplified shape, or applying a coating to functionalize the surface of a fully formed element with specific geometry.

LENS technology has many advantages compared to other 3D printing methods. The basic one is the lack of restrictions in the powder feeding process. Some LENS models can realize five-axis control. Additionally, the typical build volume is comparatively large and can be 900 × 1500 × 900 mm (LENS 1500, 2018). However, the truly defining feature of the LENS process is the ability to fabricate with multiple materials and create functionally graded materials, owing to the multiple powder feed lines. Due to the high temperatures generated in the printing process, it is possible to produce products from materials with a high melting point. This method also makes it possible to produce complex porous structures. The method also makes it possible to reprint used parts, as there is no need to start printing on a flat platform. The LENS method also has disadvantages. Due to the possibility of deposition of partially melted powder particles on the printing surface, a high surface roughness is achieved. In the case of the Ti-6Al-4V material, increasing the surface roughness reduces the fatigue strength [15]. The technology is used to produce parts in industries such as medicine and aviation [16,17,18].

The authors undertook research on the Ti-6Al-4V alloy because, due to its properties, it is more often used in the production of implants than pure Ti.

Many scientific works have been devoted to assessing the influence of hot isostatic pressing on mechanical properties and microstructures [15,16,17,18,19,20,21,22,23,24,25,26,27,28,29]. In these papers the impact of the hot isostatic pressing process on samples produced, for example, by the selective laser melting (SLM) method [11] or powder metallurgy for orthopedic and dental applications was evaluated. The results of tests on samples made using the SLM method revealed an increase in the density of the tested material. The best results were obtained for samples subjected to the HIP process at a temperature of 910 °C and a pressure of 130 MPa [9]. Research conducted at the German University of Applied Sciences Aschaffenburg on implants made of the titanium alloy Ti-6Al-4V provided interesting results. It turned out that heat treatment in the form of hot isostatic pressing increased the tensile strength of the tested samples by 15% and increased the plasticity of the samples by an impressive 53% [25]. An increase in the fatigue strength of samples printed after the HIP process was also demonstrated [30].

The cited works did not assess the mechanical properties of samples produced using the LENS method and did not check whether changing the direction of printing the samples would also have a significant impact.

In additive technology using the LENS method, an important aspect is the possibility of printing in two directions. This gives great possibilities as to the shape of printed elements. However, the question arises whether the products will have the same strength regardless of the printing direction. This issue is poorly analyzed in the literature reports compared to, for example, various heat treatment methods [31,32]. According to these hesitations, the authors decided to check whether the printing direction of Ti-6Al-4V samples affects the change in mechanical properties. Do the mechanical properties of samples subjected to the HIP process solely depend on the adopted pressure values (500, 1000, 1500 bar) or also on the printing direction? Providing an answer to the above question will provide a significant contribution to the better understanding of the factors influencing the change in the properties of the Ti-6Al-4V alloy.

## 2. Materials and Methods

Within the study the used samples were made of the Ti-6Al-4V titanium alloy, whose chemical composition is shown in Table 1, using the LENS method. The samples were originally in the form of powder with a grain size ranging from 20 µm to 63 µm.

The choice of material was dictated by its medical applications. This alloy is biocompatible and the tested technique allows for the creation of spatial structures adapted to specific individual needs. However, before it is possible to make ready-made correct prints that can be implanted, the assumptions must be examined. Therefore, durability tests of the prints were performed, the aim of which was to determine the directions of changes for individual parameters.

The LENS method melted the powder and connected the material using a laser beam. Ultimately, the samples took the form of paddles measuring 65 × 10 × 2 mm. The samples were printed in two different orientations on the build plate: vertical (samples marked Y) and horizontal (samples marked X). After the printing stage, the samples were annealed at a temperature of 920 °C. This process lasted 4 h and was aimed at removing impurities such as inert gases (Ar) and inclusions, and removing internal stresses. After annealing, the samples were rinsed twice in an ultrasonic bath using waves with a frequency of 37 kHz and then dried. Both the rinsing and washing processes took 60 min. The final stage was the mechanical grinding and polishing of each sample surface. In the next stage, the samples were divided into two groups. One group of samples was subjected to hot isostatic pressing, while in the second group, this step was omitted (X1 ÷ X6, Y1 ÷ Y6). The samples subjected to the HIP process were further divided into three groups. Each group differed in the set pressure that prevailed in the chamber during heat treatment. Table 1 contains detailed parameters of the HIP process for individual groups.

The hot isostatic pressing process took place in a special chamber located in an appropriate furnace (Figure 1). The vessel with the sample inside was subjected to a high temperature and high isostatic pressure (Table 2). The pressure in the entire chamber was constant and affected the material in the same way from every direction.

**Figure 2:**
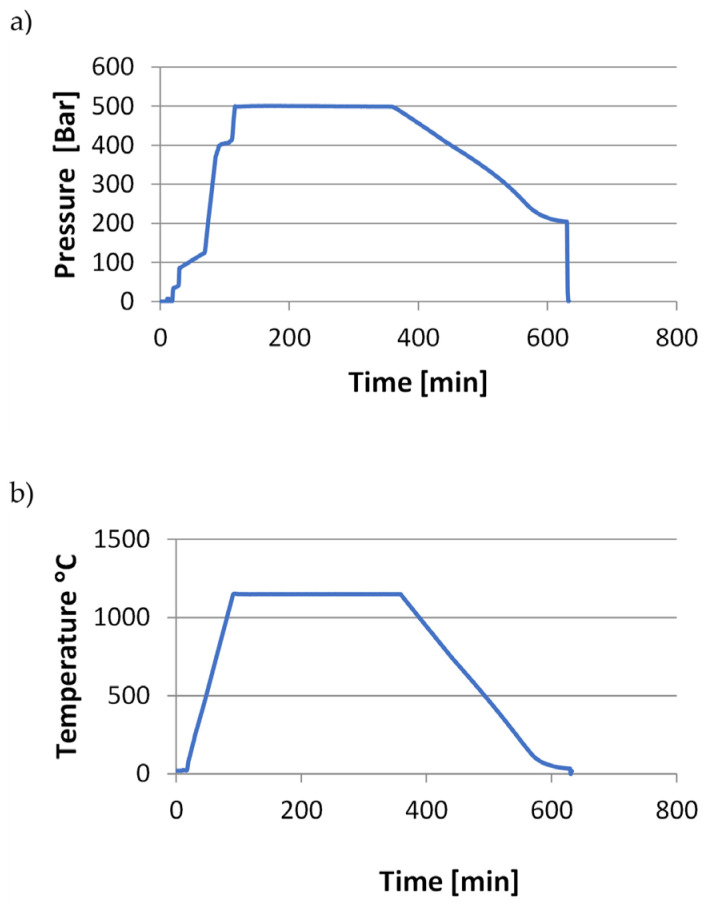
Process curves for the samples (X1.1 ÷ X1.4, Y1.1 ÷ Y1.3): (**a**) pressure changes over time, (**b**) temperature changes over time.

**Figure 3:**
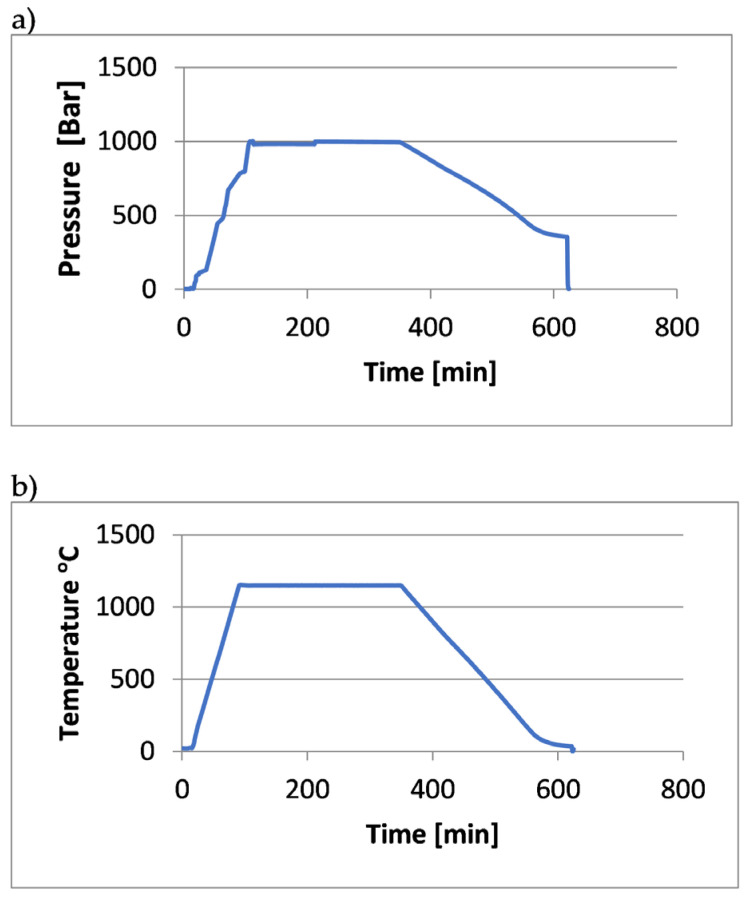
Process curves for the samples (X2.1 ÷ X2.4, Y2.1 ÷ Y2.4): (**a**) pressure changes over time, (**b**) temperature changes over time.

**Figure 4:**
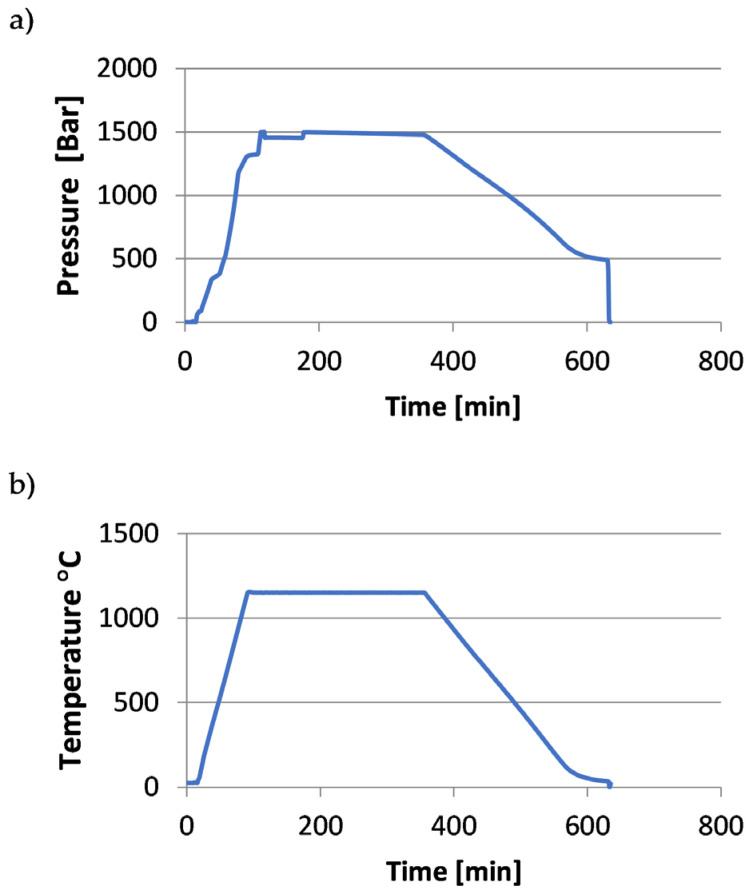
Process curves for the samples (X3.1 ÷ X3.2, Y3.1 ÷ Y3.3): (**a**) pressure changes over time, (**b**) temperature changes over time.

Figure 2, Figure 3 and Figure 4 show the course of the entire HIP process for the three groups of tested samples.

**Figure 5:**
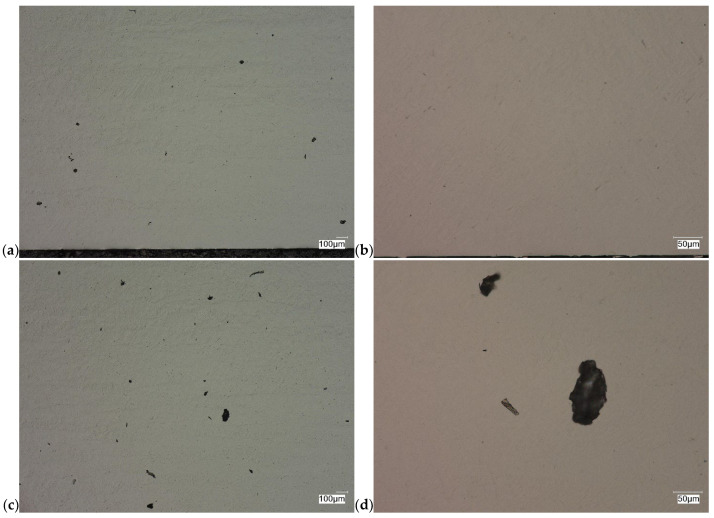
Microscopic view of the surface of sample X, unetched state: (**a**) 100× edge, (**b**) 500× edge, (**c**) 100× central part, (**d**) 500× central part.

**Figure 6:**
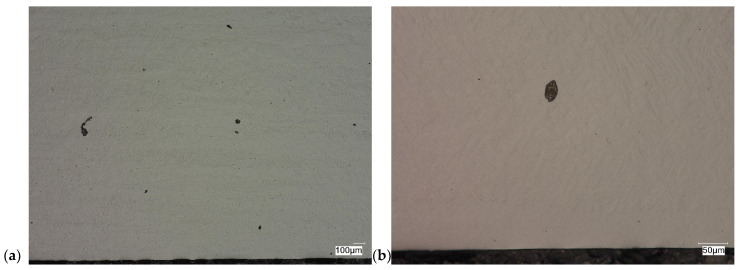

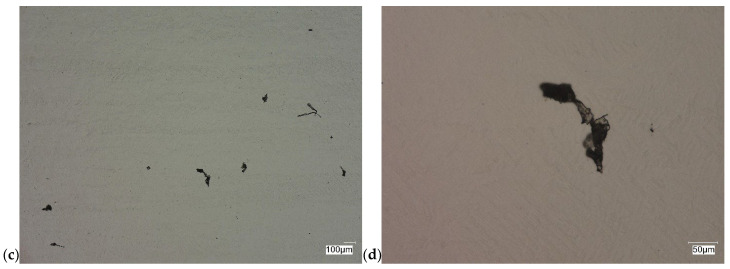
Microscopic view of the surface of sample Y, unetched state: (**a**) 100× edge, (**b**) 500× edge, (**c**) 100× central part, (**d**) 500× central part.

**Figure 7:**
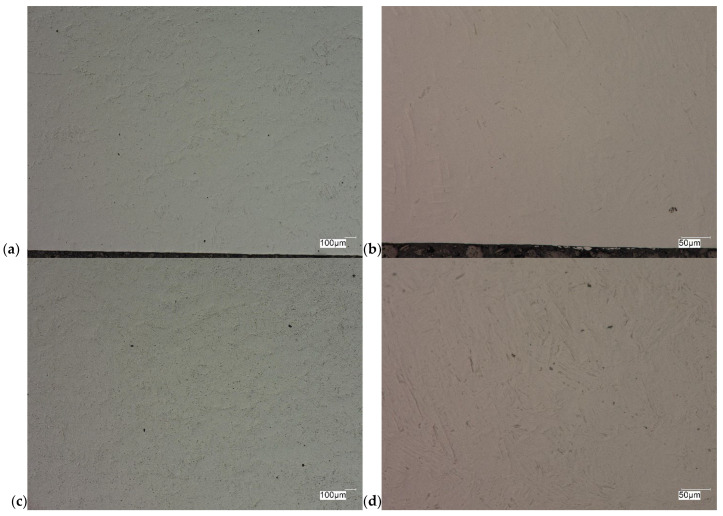
Microscopic view of the surface of sample XX, unetched state: (**a**) magnification 100× edge, (**b**) 500× edge, (**c**) 100× central part, (**d**) 500× central part.

**Figure 8:**
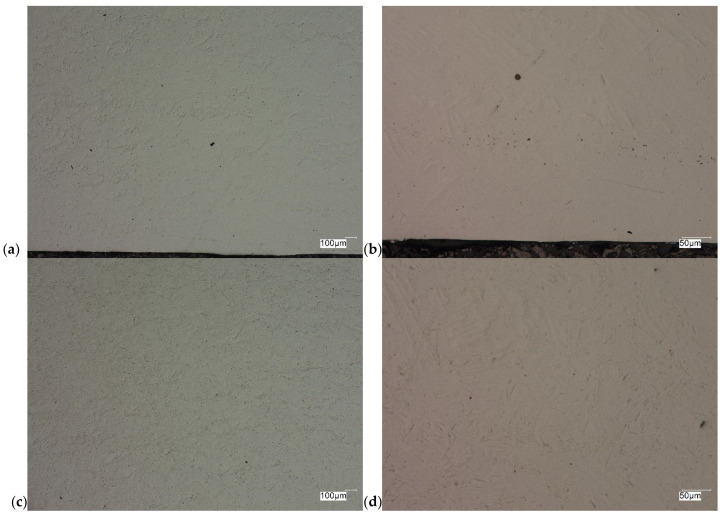
Microscopic view of the surface of sample YY, unetched state: (**a**) 100× edge, (**b**) 500× edge, (**c**) 100× central part, (**d**) 500× central part.

**Figure 9:**
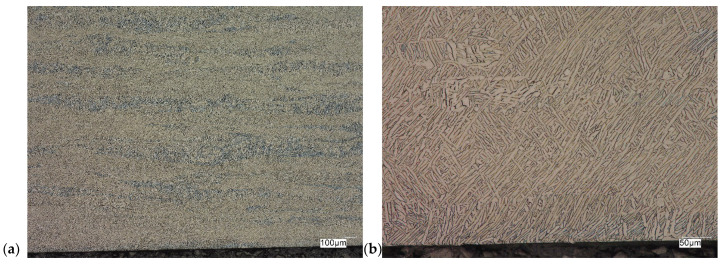

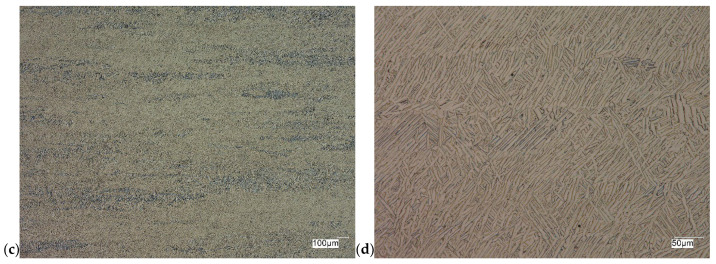
Microscopic view of the surface of sample X, etched state: (**a**) 100× edge, (**b**) 500× edge, (**c**) 100× central part, (**d**) 500× central part.

**Figure 10:**
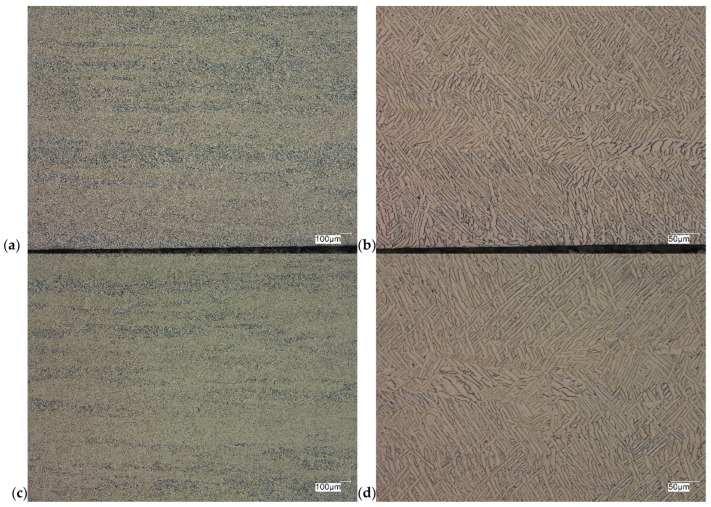
Microscopic view of the surface of sample Y, etched state: (**a**) 100× edge, (**b**) 500× edge, (**c**) 100× central part, (**d**) 500× central part.

**Figure 11:**
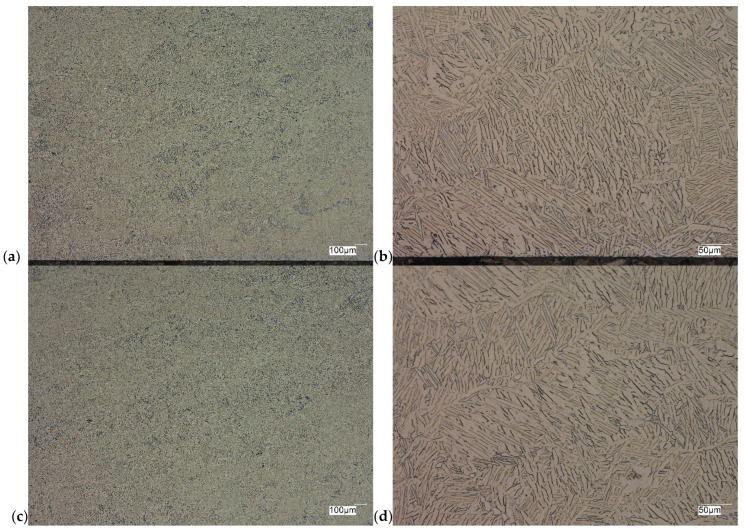
Microscopic view of the surface of sample XX, etched state: (**a**) 100× edge, (**b**) 500× edge, (**c**) 100× central part, (**d**) 500× central part.

**Figure 12:**
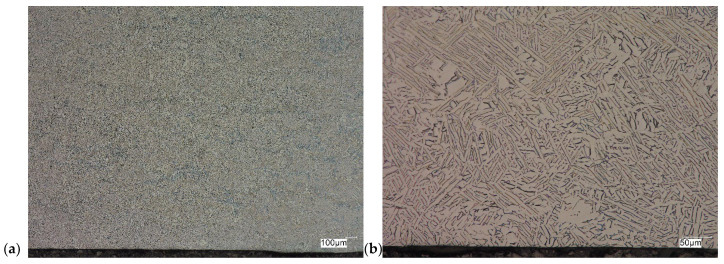

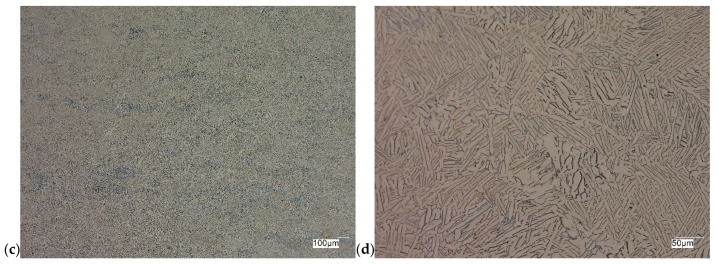
Microscopic view of the surface of sample YY, etched state: (**a**) 100× edge, (**b**) 500× edge, (**c**) 100× central part, (**d**) 500× central part.

The evaluation of the microstructure in the unetched and etched state of the samples after printing and after the HIP process is presented in Figure 5, Figure 6, Figure 7, Figure 8, Figure 9, Figure 10, Figure 11 and Figure 12. The analysis was performed using a Keyence (Osaka, Japan) VHX 7000 series optical microscope. The studies were carried out in a bright field and the sample preparation technique was standard, using diamond pastes. In order to reveal the microstructure of the material, the samples were etched with titan 2 reagent (HNO_3_ 2 mL, HF—3 mL, H_2_O—96 mL).

Prior to the HIP process, the samples exhibited the presence of monstrosity, most notably in the central region. These pores ranged in size from approximately 15 to 100 µm. They are the effects of the manufacturing process and the high reactivity of titanium. Their number decreases significantly towards the edge of the samples, which should probably be related to faster heat dissipation in these zones. Sample Y displays porosities with markedly more irregular shapes, particularly in the central region of the sample. The morphology and characteristics of these porosities suggest that they result from incomplete welding or gas entrapment at the grain boundaries.

As can be observed after the HIP process, basically all pores were closed. This is due to the long-term effect of temperature and pressure in the volume of the device chamber. This is thanks to the use of Pascal’s law, i.e., an even distribution of the pressure force on each fragment. Such parameters facilitate the occurrence of diffusion, which in consequence leads to the closure of porosity. What is also interesting is that the high reactivity of the tested alloy, combined with the significant energy supplied in the HIP process, allows for the initial disclosure of the microstructure, which will be confirmed in the photos of the etched samples presented in Figure 9, Figure 10, Figure 11 and Figure 12.

The Ti-6Al-4V alloy is a two-phase alloy with a structure (α + β), used, among others, for orthopedic implants and bone fixation elements. As can be seen in the state after printing, samples X and Y, the material is characterized by a typical bi-modal microstructure (duplex), consisting of α grains in a lamellar matrix (α + β). An important feature is the clear ordering of the separation in accordance with the directions of layer deposition. Very uniform grain sizes are also visible in both the central and edge parts. After the HIP process, with the increase in temperature and pressure (processing parameters), the average grain size and the volume fraction of the phase mixture (α + β) increase. The randomness of grain orientation in space also increases, which causes a change in the form of an observed decrease in strength properties.

As can be observed on the surface of the sample, not only are there no porosities in the material visible, but also grain boundaries are visible (even though the sample was not etched) with characteristic visible arrangements in the printing direction.

**Figure 13:**
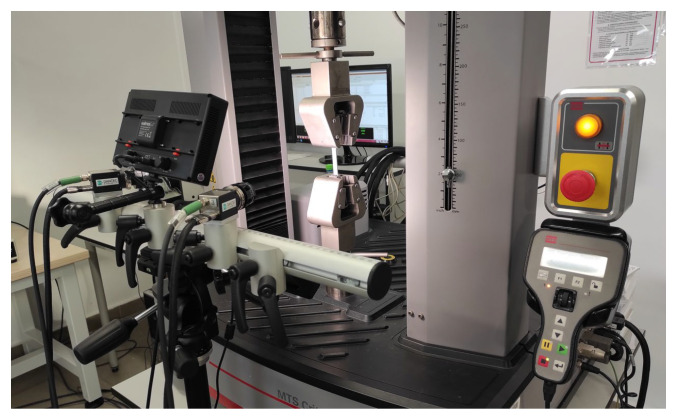
Strength testing machine and digital image correlation system.

Strength tests were carried out for both categories of samples (after and without the HIP process). The strength of the samples was assessed by performing a static tensile test at a speed of 5 mm/min using the MTS Criterion Model 43 testing machine (MTS Systems, Eden Prairie, MN, USA) and the Dantec Q400 digital image correlation system (Dantec Dynamics, Ulm, Germany) (Figure 13). A 30 kN force sensor was used for the tests.

**Figure 14:**
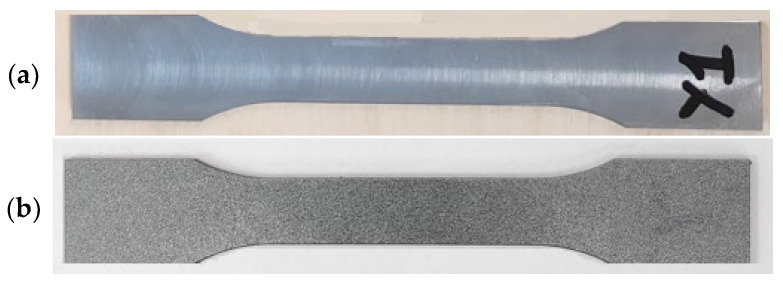
Example of a test sample: (**a**) before painting, (**b**) after painting.

The digital image correlation system is an optical measurement method for determining the distribution of object displacements and determining the 3D deformation field. This is a fully non-contact method in which the contour measurement, displacement and deformation of the material are determined during the measurements (using a testing machine). To guarantee the accuracy of the measurements, the system was calibrated using a dedicated calibration plate with characteristic points reflecting the axes of the coordinate system. The digital image correlation system requires appropriate preparation of samples for testing. The surface is covered with matte white paint. The matte tint was intended to prevent light reflections that could distort the recorded image during testing (Figure 14). After the white layer dried, additional black paint in the form of powder was applied to the samples, creating dots on their surface.

The sample was then mounted in special holders of the testing machine, ensuring the stable and axial positioning of the sample during the static tensile test. The sample was then deformed in the direction of the longitudinal axis under quasi-static conditions at a speed of 5 mm/min. The results were recorded by a digital image correlation system at a frequency of 10 Hz. The test was carried out until the sample’s failure.

## 3. Results and Discussion

The values obtained during the tests, such as the maximum force and stress at break, were read from a testing machine, while the strain value in two mutually perpendicular directions were calculated based on Young’s modulus obtained in the digital image correlation system. Poisson’s ratio was calculated based on the determined strain values. The obtained results were compared between groups of appropriately marked samples, and attention was paid to the division of samples subjected to the hot isostatic pressing process and samples that were not subjected to this process.

### 3.1. Results for Samples Not Subjected to the HIP Process

**Figure 15:**
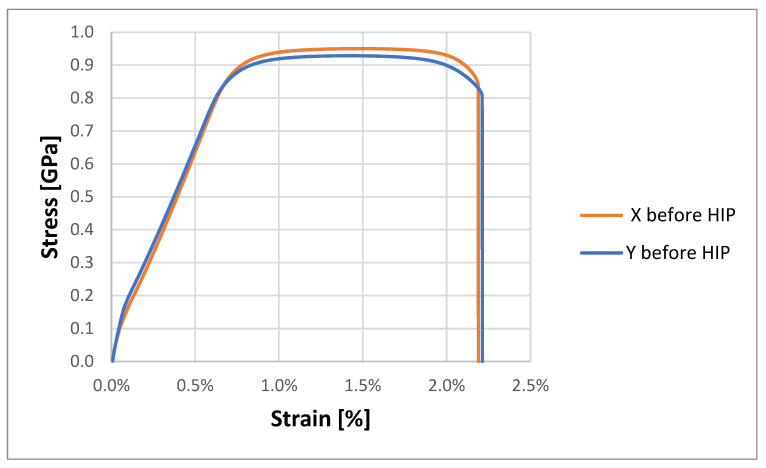
Typical stress–strain curves for samples before HIP.

The test results obtained for samples not subjected to the HIP process are presented in Table 3. Figure 15 shows the stress–strain curves for the tested samples.

Analyzing the influence of printing direction on the average values of maximum ultimate tensile stress, a higher result was observed when the print was placed horizontally, of 939.7 ± 17.89 MPa compared to 927.5 ± 4.71 MPa vertically. The average maximum force at break was higher for horizontal samples compared to vertical ones, and reached 18.79 ± 0.36 kN and 18.55 ± 0.09 kN, respectively. The difference in the obtained values is small and amounts to only 0.24 MPa. The obtained difference is smaller than the error bar value for samples X, which was 0.36 MPa. The average values of Young’s moduli were 115.4 ± 2.7 GPa for samples Y and 115.2 ± 1.6 GPa for samples X, respectively. The difference in the obtained values is very small and amounted to only 0.2 MPa. The Young’s modulus determined in the work did not show such changes depending on the direction of printing that were observed by other scientists. They obtained values of 118 GPa and 109 GPa for two printing directions [22]. The obtained strain values at break were the same and amounted to 0.28 mm/mm. The only difference observed was the value of the determined Poisson’s number for samples X = 0.34 ± 0.05 and for samples Y = 0.37 ± 0.09.

Analyzing the obtained values, no significant differences were observed between the determined parameters for horizontally printed samples compared to vertically printed samples. It can, therefore, be concluded that the direction of the bend does not have a significant impact on the change in the strength parameters of the prepared samples.

### 3.2. Results for Samples After the HIP Process

**Figure 16:**
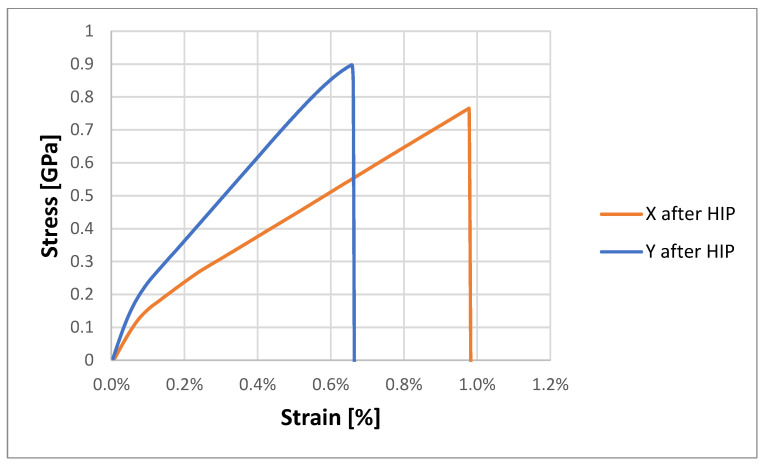
Typical stress–strain curves for samples after HIP.

The test results obtained for samples subjected to the HIP process are presented in Table 4 and Table 5. Figure 16 shows the stress–strain curves for the tested samples.

Analyzing the obtained test results for samples printed horizontally after the HIP process, the highest value of peak load was observed for samples subjected to a pressure of 1500 bar (X3.1–X3.2), which amounted to 18.67 ± 0.69 kN. The smallest one was for samples subjected to a pressure of 500 bar (X1.1–X1.4), which was 13.23 ± 2.24 kN. The highest ultimate tensile stress value of 902.03 ± 19.93 MPa was recorded for samples from the group (X3.1–X3.2), the lowest was 639.48 ± 109.77 MPa and for samples (X1.1–X1.4) subjected to a pressure of 500 bar. In the case of Young’s modulus, the highest value was obtained for samples subjected to a pressure of 1000 bar (X2.1–X2.4)—119.07 ± 2.25 GPa, and the lowest for samples (X1.1–X1.4)—114.32 ± 4, 04 GPa. Analyzing the obtained test results for horizontally printed samples after the HIP process, the highest value of peak load was observed for samples subjected to a pressure of 1500 bar (Y3.1–Y3.3), which amounted to 18.52 ± 0.14 kN. The smallest one was for samples subjected to a pressure of 500 bar (Y1.1–Y1.3), which was 14.35 ± 0.91 kN. The highest value of ultimate tensile stress, 894.98 ± 3.78 MPa, was recorded for samples from the group (Y3.1–Y3.3) and the lowest was 691.95 ± 38.19 MPa for samples (Y1.1–Y1.3), subjected to a pressure of 500 bar. The obtained values are smaller than those presented in publication [9]. The authors obtained results in the range between 1226 MPa and 955 MPa. The authors of the work used one different sample printing method (SLM). The method of sample preparation and, more precisely, their structure, is important in the final effect of using HIP, which has already been demonstrated [31]. In the case of Young’s modulus, the highest value was obtained for samples subjected to a pressure of 1000 bar (Y2.1–Y2.4)—115.4 ± 4.84 GPa, and the lowest was for samples (Y1.1–Y1.3)—114.19 ± 11, 56 GPa. The obtained values are much higher than in the case of titanium lattice structures subjected to the HIP process [25].

Analyzing the obtained test results after the HIP process, vertically printed samples were characterized by a higher average value of the plasticity pot Rp, which amounted to 122.14 ± 0.9 MPa, than horizontally printed samples, for which this value was 120.76 ± 1.49 MPa.

Analyzing the maximum peak between the group of samples not subjected to the HIP process (first group) (Table 3) and the group of samples subjected to the HIP process (second group) (Table 4), its decrease was noticed for samples after the HIP process. For samples from the first group printed horizontally, X, the average maximum peak load was 18.79 kN, and when samples were printed vertically, Y, its average value was 18.55 kN.

For samples from the second group, the average value of the maximum peak load for samples X was 16.32 kN. The highest value was obtained for samples subjected to the HIP process at a pressure of 1500 bar, 18.67 kN, while the lowest value was 13.23 kN for samples subjected to the HIP process at a pressure of 500 bar. For Y samples from the second group, the average maximum peak load is 17.05 kN. The highest value was observed in the samples heat-treated at a pressure of 1500 bar, measuring 18.52 kN, whereas the lowest value, 14.35 kN, was recorded in the samples subjected to the HIP process at a pressure of 500 bar.

Based on the above data, it can be seen that the hot isostatic pressing process resulted in an increase in the Young’s modulus value in several cases, which suggests an increase in the stiffness of the material. The highest increase in Young’s modulus of 3.8% was recorded for samples printed in the Y direction and subjected to the HIP process at a pressure of 500 bar. The greatest decrease was observed for samples printed in the Y direction subjected to the HIP process at a pressure of 1500 bar.

The isostatic pressing process worked best at a pressure of 1000 bar for samples printed in the X direction, and at a pressure of 500 bar for samples printed in the Y direction.

In the case of samples printed in the X direction, the highest Young’s modulus values of samples after the HIP process were recorded when the process pressure was equal to 1000 bar. The remaining samples from this group were insignificantly affected by the HIP process in the context of Young’s modulus.

The obtained test results showed that the hot isostatic pressing process has a significant impact on the deformation value. In each case tested, the deformation decreased by approximately 75%. The largest change of 78.6% occurred in samples printed in the X di-rection and heat treated at a pressure of 1000 bar, and in samples printed in the Y direction and subjected to a pressure of 500 bar.

Analyzing the results obtained for the conventional yield strength R_p0.2_ (Table 4), it can be seen that the values do not differ significantly for the individual groups of tested samples. Both the variable parameters of the HIP process and the printing direction did not cause significant changes in the determined values. In the case of the yield strength, there is a visible relationship between the direction of printing samples and the parameters of the HIP process. The lowest value of the yield strength was recorded for samples printed in the X direction and subjected to hot isostatic pressing at a pressure of 500 bar. The highest value of the Rp parameter was assigned to the sample printed in the Y direction, heat treated at a pressure of 500 bar.

The yield strength could not be recorded for samples Y2.1–Y3.3. In the case of samples printed in the X direction, a relationship is visible: the higher the pressure of the HIP process, the higher the yield strength.

**Figure 17:**
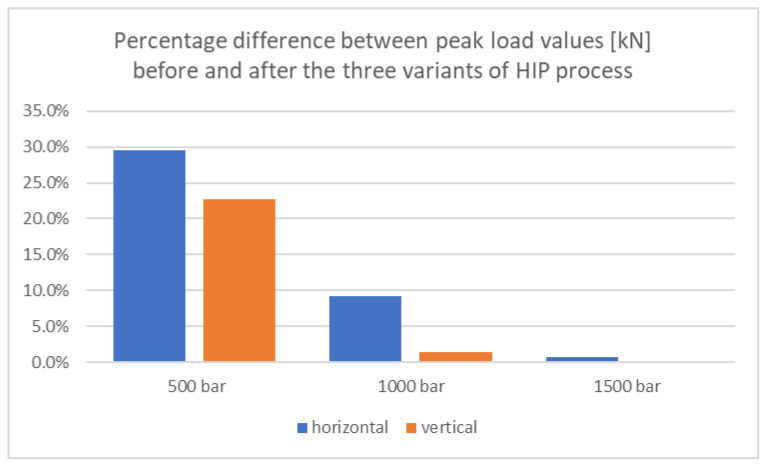
Summary of percentage differences for peak load values before and after the HIP process for three applied pressure values.

**Figure 18:**
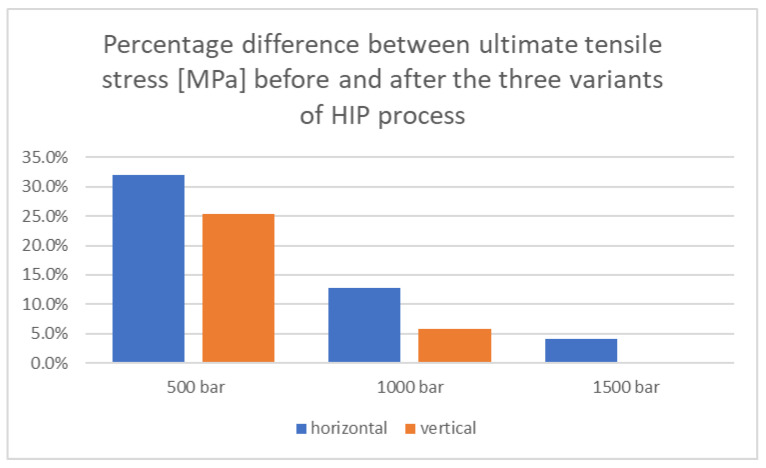
Summary of percentage differences for ultimate tensile stress values before and after the HIP process for three applied pressure values.

**Figure 19:**
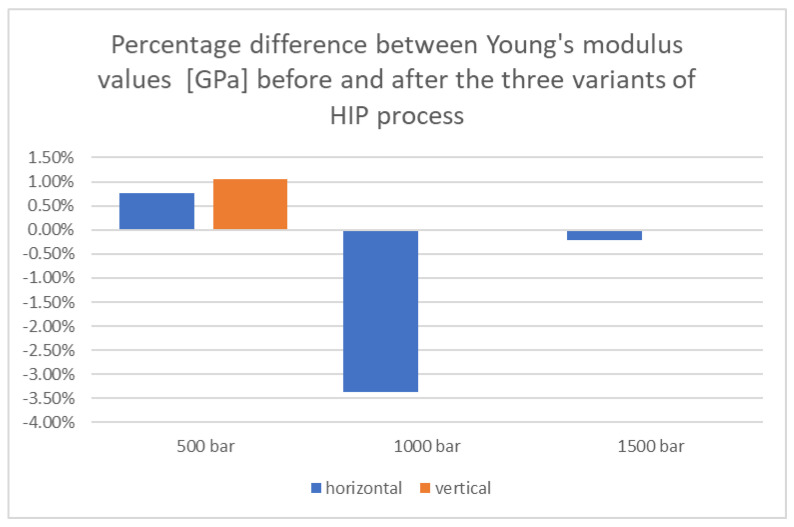
Summary of percentage differences for Young’s modulus values before and after the HIP process for three applied pressure values.

In order to better illustrate the differences that occurred between the determined parameters for samples before and after the HIP process, Figure 17, Figure 18 and Figure 19 present the calculated percentage differences between the values for samples before and after the HIP process.

The results show that the isostatic pressing process reduces the value of the maximum force at break. It was noticed that the lower the pressure of the pressing process, the greater the decrease in the maximum peak load value.

The largest percentage differences were observed for samples printed horizontally and subjected to a pressure of 500 bar, which was 29.6%, while the smallest differences were observed for samples printed horizontally and subjected to a pressure of 1500 bar, which was only 0.03%.

In the case of percentage differences for ultimate stress, the situation is similar. The largest percentage differences were observed for samples printed horizontally and subjected to a pressure of 500 bar, which was 31.9%, while for samples printed vertically and subjected to a pressure of 1500 bar, no percentage differences were observed. For a pressure of 1000 bar, samples printed horizontally showed a difference in the ultimate tensile stress value of 12.8%, while for samples printed vertically, the difference was 5.8%.

In the case of Young’s modulus, the largest percentage differences were observed for samples printed horizontally and subjected to a pressure of 1000 bar, which were 3.36%. In the case of vertically printed samples, percentage differences practically did not occur.

## 4. Conclusions

The paper evaluates the influence of manufacturing and finishing parameters on a biocompatible material, specifically the Ti-6Al-4V alloy. While these studies are preliminary, they have already provided valuable insights into the potential changes in material properties. This is particularly important, as 3D printing technology enables the fabrication of customized prostheses and even damaged tissue fragments. From the perspective of the potential applications of such elements, it is essential to determine the positive or negative impacts of various finishing techniques.

In this study, the influence of hot isostatic pressing (HIP) temperature and pressure on the mechanical properties of the Ti-6Al-4V titanium alloy, produced using the laser engineering net shaping (LENS) method, was assessed. Additionally, the study explored whether the orientation of printing in the LENS process affects the obtained strength properties. Based on the experimental results, the following conclusions can be drawn: the direction of forming the Ti-6Al-4V alloy using the LENS method does not significantly affect the tensile strength properties of the material. The recorded variations in strength were within the margin of error. A comparison of results from samples before and after the HIP process indicated that the process influenced the material’s properties. Specifically, higher applied pressures led to higher peak load values. The highest values of Young’s modulus were achieved at a pressure of 1000 bar, while the highest ultimate tensile strength (UTS) values were recorded at 1500 bar. The HIP process thus enables the control of both the strength and plasticity of the material. However, it was also observed that variations in HIP parameters significantly affect the final mechanical properties of the samples.

Depending on the applied pressure, some material properties improved while others deteriorated, an outcome that was not anticipated. Typically, the HIP process enhances the strength properties of the materials forming method [6,16,19,20], but this pattern is not always observed. Future research should investigate similar samples, incorporating variations in temperature and a broader range of pressure values during the HIP process. These studies may help establish optimal HIP parameters to improve the strength properties of samples fabricated using the LENS method.

## Figures and Tables

**Table 1:** Chemical composition of the used material.

	Ti	Al	V	Fe	C	N	H	O_limit_	O_typical_
Grade 5	Bal.	5.5–6.75	3.5–4.5	≤0.40	≤0.08	≤0.05	≤0.015	≤0.20	0.11

**Table 2:** Parameters of the hot isostatic pressing process.

Sample Number	Temperature [°C]	Pressure [Bar]	Time [min]
X1.1 ÷ X1.4	1150	500	240
Y1.1 ÷ Y1.3			
X2.1 ÷ X2.4		1000	
Y2.1 ÷ Y2.4			
X3.1 ÷ X3.2		1500	
Y3.1 ÷ Y3.3			

**Table 3:** Test results for samples without the HIP process.

Sample Number	Peak Load [kN]	Ultimate Tensile Stress [MPa]	Young’s Modulus [GPa]	Strain at Break [mm/mm]	Poisson’s Ratio
X1	18.94	946.93	-	0.027	-
X2	19	950.36	116.6	0.027	0.3
X3	18.9	945.26	114.3	0.025	0.4
X4	18.87	943.47	114	0.029	0.4
X5	18.97	948.64	113.9	0.031	0.3
X6	18.07	903.51	117.3	0.027	0.3
Average X	18.79 ± 0.36	939.70 ± 17.89	115.2 ± 1.6	0.028 ± 0.02	0.34 ± 0.05
Y1	18.52	925.75	113.8	0.024	0.3
Y2	18.58	928.81	113.2	0.027	0.4
Y3	18.63	931.34	114.3	0.030	0.5
Y4	18.56	928.11	113.8	0.032	0.3
Y5	18.64	932.13	117.6	0.027	0.3
Y6	18.383	919.16	119.9	0.03	0.4
Average Y	18.55 ± 0.09	927.55 ± 4.71	115.4 ± 2.7	0.028 ± 0.03	0.37 ± 0.09

**Table 4:** Test results for samples after the HIP process.

Sample Number	Peak Load [kN]	Ultimate Tensile Stress [MPa]	Young’s Modulus [GPa]	Strain at Break [mm/mm]	Poisson’s Ratio
X1.1	10.43	501.04	113.58	0.07	0.35
X1.2	15.78	765.04	110.5	0.1	0.31
X1.3	13.91	669.74	120.03	0.05	0.30
X1.4	12.79	622.12	113.17	0.04	0.25
Average (X1.1–X1.4)	13.23 ± 2.24	639.48 ± 109.77	114.32 ± 4.04	0.07 ± 0.03	0.32 ± 0.03
X2.1	15.93	776.87	119.35	0.06	0.33
X2.2	17.57	831.51	117.24	0.06	0.41
X2.3	17.36	831.15	117.53	0.06	0.32
X2.4	17.42	837.29	122.14	0.06	0.28
Average (X2.1–X2.4)	17.07 ± 0.77	819.21 ± 28.36	119.07 ± 2.25	0.06 ± 0	0.34 ± 0.05
X3.1	18.187	887.94	113.88	0.07	0.37
X3.2	19.156	916.12	117.01	0.08	0.42
Average (X3.1–X3.2)	18.67 ± 0.69	902.03 ± 19.93	115.45 ± 2.21	0.08 ± 0.01	0.4 ± 0.04
Average X	16.32 ± 2.80	786.91 ± 134.22	116.28 ± 2.48	0.07 ± 0.01	0.35 ± 0.04
Y1.1	14.48	695.98	126	0.06	0.29
Y1.2	13.39	651.91	102.9	0.06	0.36
Y1.3	15.19	727.97	113.67	0.06	0.42
Average (Y1.1–Y1.3)	14.35 ± 0.91	691.95 ± 38.19	114.19 ± 11.56	0.06	0.36 ± 0.07
Y2.1	18.02	866.15	115.97	0.07	0.32
Y2.2	18.2	868.53	121.99	0.08	0.34
Y2.3	17.94	860.62	111.12	0.07	0.33
Y2.4	18.98	898.72	112.52	0.1	0.34
Average (Y2.1–Y2.4)	18.28 ± 0.47	873.50 ± 17.13	115.4 ± 4.84	0.08 ± 0.01	0.33 ± 0.01
Y3.1	18.53	897.71	131.34	0.07	0.43
Y3.2	18.66	896.56	105.84	0.07	0.29
Y3.3	18.37	890.67	108.5	0.07	0.28
Average (Y3.1–Y3.3)	18.52 ± 0.14	894.98 ± 3.78	115.23 ± 14.02	0.07	0.33 ± 0.08
Average Y	17.05 ± 2.34	820.14 ± 111.54	114.94 ± 0.66	0.07 ± 0.01	0.34 ± 0.01

**Table 5:** Average test results for samples after the HIP process.

Samples After the HIP Process	R_p0.2_ [MPa]	R_p_ [MPa]
(X1.1–X1.4)	123.68 ± 0.91	119.33 ± 3.1
(X2.1–X2.4)	122.76 ±1.57	120.65 ± 5.44
(X3.1–X3.2)	123.53 ± 1.8	122.31 ± 0.2
Average X	123.32 ± 0.49	120.76 ± 1.49
(Y1.1–Y1.3)	123.71 ± 0.49	122.82 ± 1.49
(Y2.1–Y2.4)	-	-
(Y3.1–Y3.3)	122.79 ± 0.35	121.45 ± 1.32
Average Y	123.25 ± 0.65	122.14 ± 0.9

## Data Availability

Data is contained within the article. Dataset available on request from the authors.
